# Remaining as a Clinical Doctor in a Smaller Institute after Retirement: A Personal View

**DOI:** 10.31662/jmaj.2023-0169

**Published:** 2024-02-09

**Authors:** Shigeki Matsubara

**Affiliations:** 1 Department of Obstetrics and Gynecology, Jichi Medical University, Tochigi, Japan; 2 Department of Obstetrics and Gynecology, Koga Red Cross Hospital, Koga, Japan; 3 Medical Examination Center, Ibaraki Western Medical Center, Chikusei, Japan

**Keywords:** clinician, doctors’ career, university, professor, retirement

## Abstract

Clinical doctors with overwhelming workloads at university or center hospitals may not have sufficient time to allocate for each patient or to consider each patient’s personal condition. Retirement may be a good chance to make a new start by becoming a clinical doctor in a smaller institute. Becoming a clinical doctor in a smaller institute may give you satisfaction and happiness different from being a university clinical doctor. I believe that after retirement from a university or big hospital, older clinical doctors should continue to participate in clinical practice for as long as they wish. This may be one of the solutions for providing clinical doctors in the current and coming super-aged Japan.

There are various personal options on what clinical doctors should do after retiring from a university or center hospitals ^(1)^. Here, I wish to state my view, which is solely based on my own experience. I conclude that after retirement, clinical doctors should continue to participate in clinical practice for as long as they wish.

I wish to write this view for clinical doctors, doctors who participate in clinical practice. Retirement system varies from country to country. Here, I describe things based on Japanese situations; however, the present view may be of some use for clinical doctors of not only Japan but also many other countries.

In Japan, many university or center hospitals have regulations; one of them is that staff should retire at the age of 60 or 65. I retired from being a university professor of an Obstetrics and Gynecology Department at the age of 65. I now work as a clinical doctor at two smaller institutes every morning from Monday to Saturday. After this, I study medicine and write medical articles. I, as a visiting professor, also help younger staff to write papers. Approximately 4 years have passed since I became a clinical doctor in smaller institutes. I now feel that I have become friendlier to and possibly more highly regarded by my patients. This gives me satisfaction and happiness. The following four factors make me feel so:

First, I can take more time with each patient. In my university era, I had various jobs other than that of a clinical doctor. My tight schedule did not allow me to spend sufficient time to consider each patient’s personal situation or feelings. Now, I have sufficient time for each patient: Nothing forces me to rush. I carefully listen to patients’ voices and often sympathize with their conditions. This situation causes less stress and gives more satisfaction to both patients and the doctor (myself).

Second, I can take care of patients from their first visit to the follow-up completion, which satisfies me. In my university era, I mainly dealt with new patients, made diagnoses, and decided overall treatment strategies. I performed several lifesaving surgeries. These are important jobs; however, after diagnosing conditions or surgeries, I told staff what to do. I did not follow patients’ entire courses. Now, I am the only gynecologist in my institute. Thus, there is no way to leave a patient’s follow-up to someone else. I feel more responsibility for each patient, which, in turn, gives me marked satisfaction as a clinical doctor.

Third, I can afford much time to ameliorate each patient’s suffering, even though the condition is generally considered less grave. For example, the target diseases have changed from obstetric life-threatening hemorrhage to urinary incontinence or hot flashes. However, I believe that the treatment of the latter is no less important than the former. Accepting patients with all kinds of complaints gives me satisfaction.

Fourth, I can also remain active in the scientific world. Becoming a clinical doctor in a smaller institute gave me more chances to encounter patients with subtle complaints, whom university doctors rarely encounter. Some important clinical lessons are often involved. Subtle complaints proved to be signs of unexpected disorders: (i) slight abdominal pain in an adolescent girl ^(2)^, (ii) slight abdominal distension ^(3)^, or (iii) vaginal odor in a postmenopausal woman ^(4)^. These conditions were finally diagnosed as (i) hymenal atresia, (ii) urinary retention, and (iii) pyometra, respectively. I learned clinical lessons from these cases, which have been published ^(2), (3), (4)^ in international journals such as *BMJ* ^(2)^. Being a clinical doctor in a smaller institute gave me viewpoints different from those of university clinical doctors, enabling me to recognize the novelty of these cases.

Please permit me to touch on my personal story. I used to be the only clinical doctor on a remote island when I was 27-29 years old. A close personal relationship with patients was naturally forged. I will never forget the experience; when I left the island, approximately one-third of the island residents gathered to see me off to say “thank you.” I feel that I was a beloved clinical doctor.

I never regret being a university staff member: I invented lifesaving surgeries, educated younger generations, and performed both basic and multidisciplinary clinical studies. Now, four decades after my island clinical doctor’s era, I have returned to being an ordinary clinical doctor. I no longer perform lifesaving surgeries. Instead, I can take abundant time, sympathize more with patients, and fully follow-up patients. Retiring from a university does not mean retiring from research or publishing. These experiences gave me great satisfaction, happiness, and ease.

I must add four issues. First, I previously wrote a short letter regarding some aspects of older clinical doctors ^(5)^. I here describe things from different viewpoints and made no repetition. Second, I never intend to compare clinical doctors’ overall happiness or satisfaction between a university and a smaller institute. They have different responsibilities and social and medical aspects. Third, I do not know how many clinical doctors working at university or center hospitals continue to practice at smaller institutions after retirement. I believe that some clinical doctors do so, and they may feel the same as myself. However, to my knowledge, no, or if any few, publication focuses on this issue. This is the reason why I wrote my present view. Fourth, I may have emphasized that clinical doctors in university hospitals are very busy. I understand that many clinical doctors working in a small clinic or any other settings are also very busy, similar to those of university or center hospitals. I wrote the present view solely based on my experience and I believe that the present description may be of some help for clinical doctors near, at, or after retirement from university or center hospitals.

Japan has now become a “super-aged country,” and this also holds true for the Japanese medical world. The situation is expected to become worse in the near future. The same may hold true to many other countries. I believe that older clinical doctors’ power and knowledge should be fully utilized. Irrespective of retirement from university or center hospitals, older doctors should remain clinical doctors for as long as they wish. Older clinical doctors still can do something for patients, the medical world, and society.

## Article Information

### Conflicts of Interest

None

### Acknowledgement

I thank Teppei Matsubara (Massachusetts General Hospital, Harvard Medical School, USA) and Daisuke Matsubara (Jichi Medical University, Japan) for their help.

### Author Contributions

S. Matsubara: identification of the significance and manuscript writing.

### Approval by Institutional Review Board (IRB)

Not applicable

### Patient Anonymity

Not applicable

### Informed Consent

Not applicable

### Data Availability

Data sharing is not applicable to this article, as no new data were created or analyzed in this study.
